# Potential Human Health Hazard of Post-Hurricane Harvey Sediments in Galveston Bay and Houston Ship Channel: A Case Study of Using *In Vitro* Bioactivity Data to Inform Risk Management Decisions

**DOI:** 10.3390/ijerph182413378

**Published:** 2021-12-19

**Authors:** Zunwei Chen, Suji Jang, James M. Kaihatu, Yi-Hui Zhou, Fred A. Wright, Weihsueh A. Chiu, Ivan Rusyn

**Affiliations:** 1 Interdisciplinary Faculty of Toxicology, Texas A&M University, College Station, TX 77843, USA; zchen@cvm.tamu.edu (Z.C.); sjang@cvm.tamu.edu (S.J.); wchiu@cvm.tamu.edu (W.A.C.); 2 Civil & Environmental Engineering and Ocean Engineering, Texas A&M University, College Station, TX 77843, USA; jkaihatu@civil.tamu.edu; 3 Biological Sciences and Statistics, North Carolina State University, Raleigh, NC 27695, USA; yihui_zhou@ncsu.edu (Y.-H.Z.); fred_wright@ncsu.edu (F.A.W.)

**Keywords:** new approach methods (NAMs), environmental mixtures, disaster research

## Abstract

Natural and anthropogenic disasters may be associated with redistribution of chemical contaminants in the environment; however, current methods for assessing hazards and risks of complex mixtures are not suitable for disaster response. This study investigated the suitability of *in vitro* toxicity testing methods as a rapid means of identifying areas of potential human health concern. We used sediment samples (n = 46) from Galveston Bay and the Houston Ship Channel (GB/HSC) areas after hurricane Harvey, a disaster event that led to broad redistribution of chemically-contaminated sediments, including deposition of the sediment on shore due to flooding. Samples were extracted with cyclohexane and dimethyl sulfoxide and screened in a compendium of human primary or induced pluripotent stem cell (iPSC)-derived cell lines from different tissues (hepatocytes, neuronal, cardiomyocytes, and endothelial) to test for concentration-dependent effects on various functional and cytotoxicity phenotypes (n = 34). Bioactivity data were used to map areas of potential concern and the results compared to the data on concentrations of polycyclic aromatic hydrocarbons (PAHs) in the same samples. We found that setting remediation goals based on reducing bioactivity is protective of both “known” risks associated with PAHs and “unknown” risks associated with bioactivity, but the converse was not true for remediation based on PAH risks alone. Overall, we found that *in vitro* bioactivity can be used as a comprehensive indicator of potential hazards and is an example of a new approach method (NAM) to inform risk management decisions on site cleanup.

## 1. Introduction

Natural disasters such as floods and hurricanes can lead to severe damage to urbanized estuarine environments and pose potential environmental and public health risks due to the re-distribution of chemical contaminants [1]. These challenges are especially acute in areas with known legacy contaminations whereby natural and anthropogenic disasters may alter the contamination patterns and change potential hazards in unpredictable ways. One recent example of such an event is Hurricane Harvey (2017), which resulted in extreme flooding in the Houston/Galveston Bay region, a heavily industrialized area on the shores of the Galveston Bay and the Gulf of Mexico. The sediments in the Galveston Bay are known to be contaminated by various types of hazardous chemicals including polycyclic aromatic hydrocarbons (PAHs), polycyclic biphenyls (PCBs), pesticides, and heavy metals [2]. Indeed, recent studies indicated that post-Harvey, pollutants such as PAHs were redistributed in the environmental matrixes such as sediments [3,4] and soil [5,6], leading to potential new human health risks. Because disasters are emergency events, there is a pressing need to develop methods for rapid and comprehensive assessments of potential exposures to, and hazards of, complex environmental mixtures.

To better characterize the potential hazards of complex mixtures in the environment, three general approaches are most commonly pursued: (i) improvements to the analytical techniques to evaluate chemical composition of the mixtures, (ii) application of new *in vitro* test methods to evaluate potential human and environmental hazards, and (iii) creation of *in silico* models for predicting the composition and effects of mixtures [7]. Increased sensitivity and broadening of the applicability domain of analytical techniques through untargeted and high-resolution mass spectrometry allows for the characterization of complex mixtures [8]. Indeed, the successful application of untargeted analytical methods to disaster research response has been demonstrated recently for events that occurred in the Houston/Galveston Bay area [9,10]. Similarly, biological assays show the potential to facilitate identification of mixtures that may pose human and/or environmental health risks [11,12,13]. Finally, computational approaches have also been developed, many of them based on utilizing the information from both exposure characterization and bioactivity measurements, to identify the chemicals of concern in mixtures that may be the drivers to the overall bioactivity [14,15,16].

Still, knowledge gaps exist in whether the combination of chemical measurements and biological *in vitro* assays can improve assessment of the potential human health risks of complex mixtures, new exposures that are a product of a large-scale environmental disaster in an area of prior legacy contamination. Therefore, this project explored the utility of chemical exposure assessment and *in vitro* bioactivity data from the same post-disaster sediment samples as data inputs for potential risk management and remediation of the contaminated areas.

## 2. Materials and Methods

### 2.1. Sediment Deposition Model

**Figure 1:**
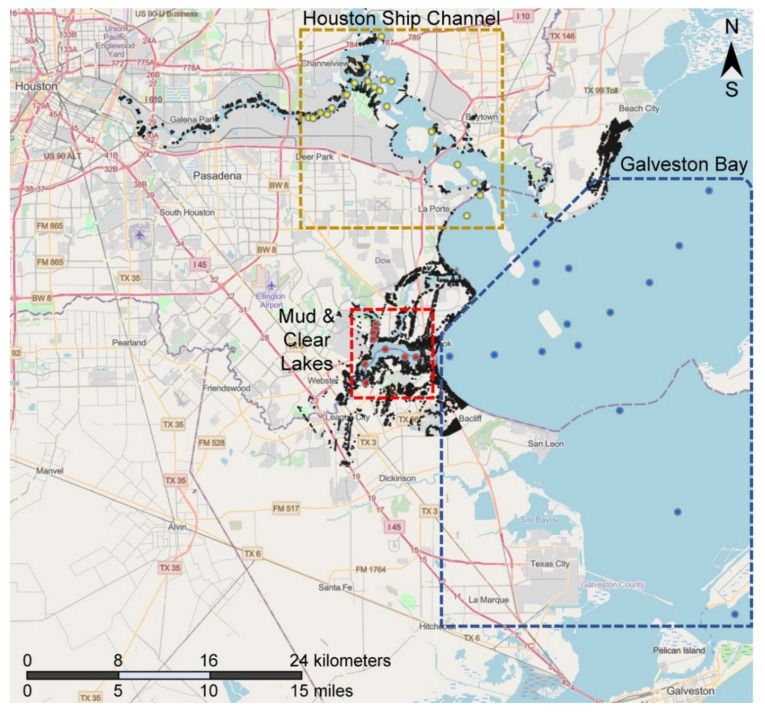
Map of the study area of Houston Ship Channel and Galveston Bay in Houston, Texas. Black dots represent areas of potential sediment deposition on shore after a major hurricane (see Section 2.1 for details). Circles represent individual sediment sampling sites, which are divided into three areas: samples from the Houston Ship Channel (yellow), Mud and Clear Lakes (red), and Galveston Bay (blue). See Supplementary Table S1 for exact coordinates of each sampling site. Background map is from ESRI/OpenStreetMap.

To demonstrate the potential for sediment deposition following a hurricane, we used Delft3D [17] to model where sediment deposition occurs following a hypothetical tropical storm scenario. We chose a “Super-Ike” storm, so named because the track and storm size characteristics were identical to 2008’s Hurricane Ike, but the wind speeds (retrieved from the HWIND database [18]) were doubled to serve as a proxy for a severe storm in a future climate scenario. Hurricane-induced surge and waves within the model were used to drive bottom sediment motion onto adjoining land using a transport algorithm within the Delft3D modeling suite. The bottom was assumed to be predominantly covered by very fine sand, with an in-place density of 2650 kg/m^3^ and a diameter of 0.2 mm. The model was configured so that only undersea sediment was allowed to move; any deposited sediment, therefore, originated in the Galveston Bay and environs. Model outputs indicating areas of potential sediment deposition in the study area are visualized in Figure 1 (black dots).

### 2.2. Chemicals and Biologicals 

Dimethyl sulfoxide (DMSO, cell-culture grade, ≥ 99%) was obtained from Santa Cruz Biotechnology (Santa Cruz, CA, USA). Cyclohexane (HPLC grade) was purchased from Fisher Scientific (Waltham, MA, USA). Positive control compounds for each cell type were obtained from Sigma-Aldrich (St Louis, MO, USA). Cellular staining materials including Hoechst 33342, MitoTracker^TM^ Orange CMTMRos, and Calcein Green AM were provided by Life Technologies (Grand Island, NY, USA). Four types of human induced pluripotent stem cell (iPSC)-derived cell types (iCell hepatocytes 2.0, catalog no. C1023; iCell neurons, catalog no. C1008; iCell cardiomyocytes, catalog no. C1106; and iCell endothelial cells, catalog no. C1114) used in these experiments were from Fujifilm Cellular Dynamics International (FCDI, Madison, WI, USA). Pooled human umbilical vein endothelial cells (HUVEC, catalog no. C2519A) were purchased from Lonza (Walkersville, MD, USA). Cell-specific culture media and supplements were obtained from the same vendors as the cells. 

### 2.3. Sediment Sample Collection, Extraction, and Bioactivity Screening 

Surface sediment samples (<5 cm depth) collection procedures are detailed in [4]. Samples were from Houston Ship Channel and Galveston Bay area (Figure 1 and Supplementary Table S1) and collected after Hurricane Harvey which occurred in late August-early September of 2017. Samples were collected in 8 oz. combusted glass jars with Teflon cap liners and were subsequently stored at −20 °C until further processing and analysis. Samples were freeze-dried before extraction for in vitro bioactivity screening. Biologically available fraction of each sediment sample was extracted using DMSO and cyclohexane as detailed elsewhere [11] using the standard method [19]. Briefly, 2 mL of cyclohexane and 2 mL of DMSO pre-equilibrated with cyclohexane at 10:1 ratio was added to 1 g of freeze-dried sediment sample and the suspension was thoroughly mixed by vortexing for at least 1 min. The DMSO fraction was collected after centrifugation (4700 rpm, 5 min) and this step was repeated. The combined DMSO fraction was used as stock solution for subsequent in vitro experiments. Vehicle control was prepared following the same procedures without adding sediment samples.

Samples were tested in a compendium of human primary or iPSC-derived cell lines from different tissues (hepatocytes, neuronal, cardiomyocytes, and endothelial) to test for concentration-dependent effects on various functional and cytotoxicity phenotypes (n = 34). We selected these cell types because many of the environmental chemicals expected to be present in tested sediments are known to be associated with hepatotoxicity, neurotoxicity, cardiotoxicity, and vascular toxicity (see literature review of the effects of Superfund priority list chemicals on different organs in [20]). We have published methods for using iPSC-derived cells [21,22,23,24,25,26,27] to assess the toxicity of the individual chemicals [20,28,29,30,31,32], whole mixtures [11,16], and complex substances [33,34]. The reasons we chose iPSC-derived cells are because: (i) these cells are more physiological than immortalized cell lines and can be derived for different tissues/organs [35]; (ii) they can be obtained from the same individual(s) to enable highly reproducible experiments [36]; (iii) despite some limitations with the degree of maturation, these cells compare well to primary cells in terms of their function and expected organ-specific toxicity [35,37,38]; and (iv) a small number of iPSC-derived cell types can be as informative about hazard and safety margins as the larger set of *in vitro* models [33], or many ToxCast bioassays [20]. Specifically, in a study of petroleum substances [33] we found that bioactivity data from four human iPSC-derived and HUVEC cells are superior to other immortalized cell lines to rank substances in a manner highly concordant with their expected *in vivo* hazard potential. In addition, when we tested 42 Superfund priority list chemicals (selected to represent a diverse set of chemical classes) using these five cell types [20], we found that each chemical had an effect in at least one cell type and no correlation in quantitative effects on each phenotype was evident among cell types, indicating cell type-specific effects. We also previously showed that the data from the five cell types are more conservative than that derived from ToxCast screening [39] because points of departure (POD) derived from high throughput *in vitro* data from the five human cell types performed well as a conservative surrogate for regulatory *in vivo* PODs and were less likely to underestimate *in vivo* potency and potential risk compared to other *in vitro*-based PODs.

### 2.4. Concentration-Response Modeling and Data Integration 

Point-of-departure (POD) values were derived from fitted curves with a nonlinear logistic function using vehicle control-scaled data for each treatment, which were defined as the dilutions where the fitted curve exceeded one standard deviation above or below the mean of vehicle-treated controls, using *R* software-based script as reported previously [21]. POD values were further converted into toxicological priority index (ToxPi) scores [40], which were inversely scaled from 0 to 1, with 0 indicating the lowest observed bioactivity (i.e., the highest POD value in a given dataset) and 1 indicating the highest observed bioactivity (i.e., the lowest POD value). The scaled POD values were then used as quantitative inputs in ToxPi Graphical User Interface for data integration and visualization. Sediment samples were grouped based on biological similarity in an unsupervised analysis, without prior knowledge of sample classification.

### 2.5. Associations of Sediment Spatial Locations and Biological/PAH Measurements 

The U.S. EPA priority 16 PAHs as well the total PAH concentrations in sediment samples were analyzed by Geochemical and Environmental Research Group at Texas A&M University as previously reported [4]. Bioactivity data (POD values for each cell type) and PAH concentrations were used for the analysis of potential correlation with spatial locations as detailed elsewhere [11]. Tests of spatial association for bioactivity or PAH data were performed using the standard Mantel approach [41], where the time dimension in the original method can be substituted with any type of multivariate outcome. The approach compares matrices of pairwise geographical distances to squared feature differences for all pairs of sampling sites [42]. For global tests using all biological or chemical features, distance matrices using all paired samples (i, j) were calculated using 1 - q_ij_, where q_ij_ is the Spearman correlation of all features. Each test was implemented in R and *p* values were obtained using 10,000 permutations, and p_adj_ were derived from multiple testing correction using the Benjamini-Hochberg procedure [43].

### 2.6. Mapping of PAH Concentrations and In Vitro Bioactivity Data 

To interpolate and visualize the distribution patterns of both PAHs and bioactivities for the tested sediment samples, kriging was performed in ArcGIS software (version 10.7.1, ESRI Inc., Redlands, CA, USA) on ToxPi scores calculated from selected cell types and on concentrations of individual and cumulative PAHs, as has been previously described [44,45]. Kriging was performed with the Spatial Analyst Tool with the default Spherical semi-variogram model. The Average Nearest Neighbor Tool, which measures the distance between each feature and its nearest neighbor’s location, was used to determine the lag size of 0.003. The total shape file for the Houston Shio Channel and Galveston Bay was taken from the Houston – Galveston Area Council GIS dataset (Available website: https://gishub-h-gac.hub.arcgis.com/, accessed date (17 December 2021))and modified to mask the Kriging results. All values were log-transformed prior to kriging and visualization.

### 2.7. Prediction between Chemical and Biological Measurements 

A multivariate penalized ridge regression procedure was performed for the prediction between chemical measurements (n = 40) and biological features (n = 34) of each sediment sample. Evaluations were performed using leave-one out cross-validation, i.e., prediction for elements in one matrix from the *ith* sample using coefficients obtained after removing the *ith* sample, to avoid overfitting. The approach involves selecting a single tuning parameter, performed to give minimum mean-squared prediction error. Final predictions were returned to the original scale by multiplying each column by the original standard deviation and adding the original mean. The details of the prediction approach and parameter tuning are detailed elsewhere [11].

### 2.8. Hazard Index and Cancer Risk Calculation for PAHs

To characterize the human health risks from PAHs using traditional risk assessment approaches, the PAH concentration of each sediment sample was used to calculate the hazard index (HI) and cancer risk (CR). To be conservative, concentrations of alkylated PAHs were added to the parent PAHs, respectively. The assessment was performed based on U.S. EPA Regional Screening Levels (RSLs) in two scenarios, e.g., recreate exposure to sediment, which includes 5 days per year and 6 hours each day, and the scenario that considers sediment as residential soil, which assumes the exposure occurs after the sediments were deposited on land. Original RSL calculation parameters were downloaded from U.S. EPA website (https://epa-prgs.ornl.gov/cgi-bin/chemicals/csl_search (accessed date (17 December 2021)) and summarized in Supplementary Files S1 and S2. The cancer risk was expressed as the number of cases per million individuals.

### 2.9. Comparing Alternative Remediation Goals

We compare two approaches for setting remediation goals. In the “traditional” approach, the remediation goal is set by requiring a HI ≤ 1 and CR ≤ 1 in a million. Specifically, this goal corresponds to a remediation “dilution factor” that represents the amount of reduction in concentration necessary to satisfy the HI or CR requirement. In the “bioactivity-based” approach, the remediation goal is set by requiring that no more than 10% of the *in vitro* endpoints are bioactive. Because the bioactivity concentration-response functions are already expressed in terms of dilution from the original extraction, this goal corresponds to the 10th percentile POD across endpoints and cell types. In each case, the “residual” risk or bioactivity is calculated to determine the extent to which the “traditional” approach is protective with respect to bioactivity and vice versa.

## 3. Results and Discussion

### 3.1. Study Area and Sample Selection Rationale

Using hydrodynamic modeling, we show that a “Super-Ike” storm can result in substantial deposition of sediments into populated areas (Figure 1, black dots), particularly along the Houston Ship Channel, in the Mud & Clear Lakes area, and along the Galveston Bay coast south of Beach City, TX. Modeled on-shore sediment deposition depths ranged from 0 to 3.4 cm, although the vast majority of deposition depths were < 0.1 cm. The modeled wind-induced surge for the Super-Ike storm (not shown) appeared to be greatest in the region of Mud Lake and Clear Lake, likely due to the lower elevations in these areas; this explains the greater density of deposited sediments in this region (Figure 1). Accordingly, the sediment samples (n = 46) that were collected throughout Galveston Bay and Houston Ship Channel areas appear qualitatively representative of sediments that may result from the deposition on land after a comparable tropical storm (Figure 1). Hurricane Ike was a relatively fast-moving storm, and this Super-Ike modeled storm shared those track characteristics. As sediment transport and morphological change have different time scales [17], one can reasonably expect even greater deposition with a slower-moving storm of similar power.

### 3.2. Bioactivity of Sediment Samples

**Figure 2:**
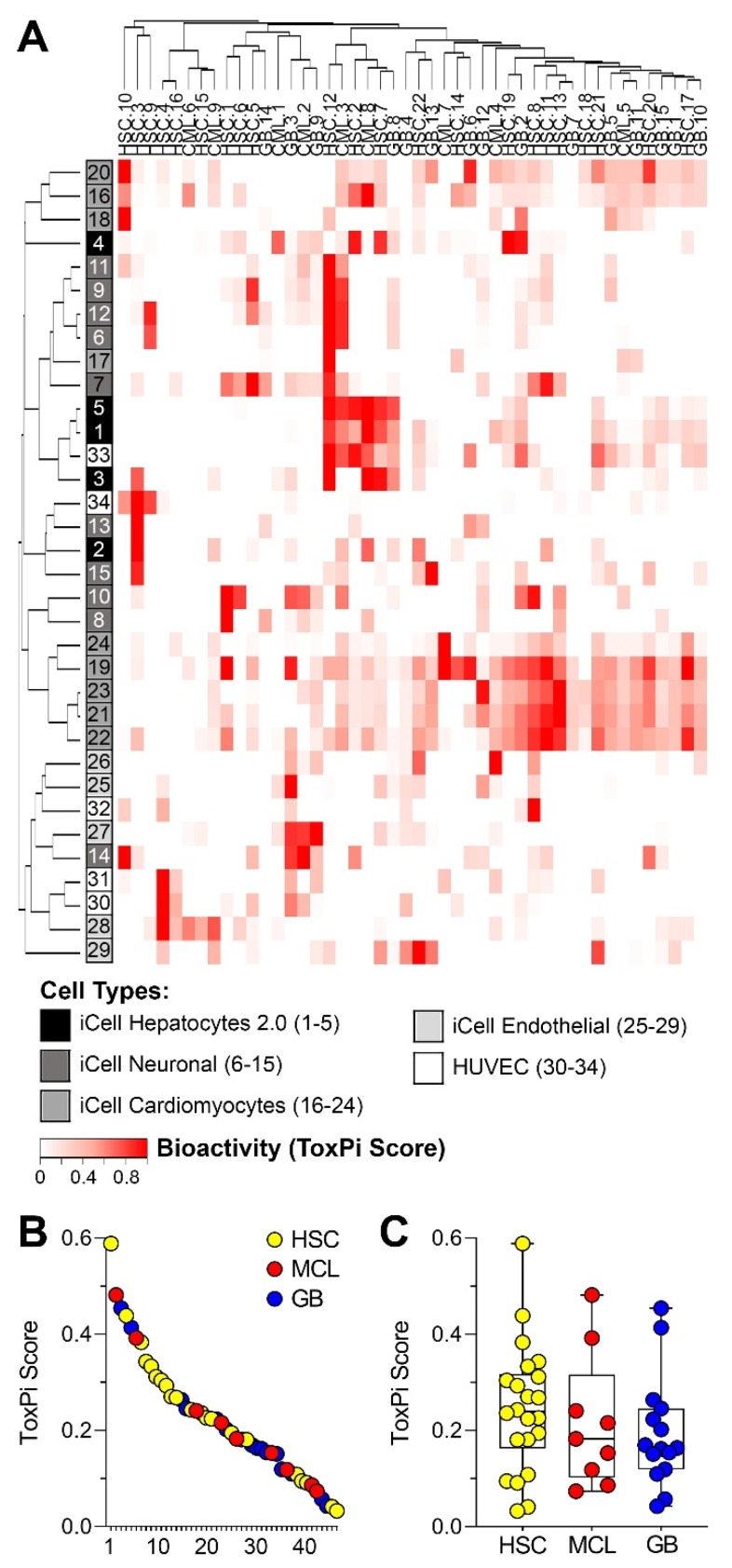
Biological profiles of sediment samples collected from the Houston Ship Channel (HSC), Mud and Clear Lakes (MCL), and Galveston Bay (GB). (**A**) Heatmap showing relative bioactivity of each sample (columns) in each cell type/phenotype (rows). Hierarchical clustering was used to organize rows and columns by similarity. See Supplementary File S3 for the data. (**B**) Ranking of the samples based on the overall ToxPi scores ranking of all samples. Samples from each study area are color coded as indicated in Figure 1. (**C**) Box and whiskers plots (line = mean, box = interquartile range and whiskers are min/max values) showing the distribution of the overall bioactivity ToxPi scores in each study area.

Quantitative estimates of *in vitro* effects of sediment extracts from a targeted set of human cell-based models and phenotypes were used to evaluate potential human health hazard of each sample (Figure 2A). Many of the samples exhibited little to no bioactivity across tested phenotypes, as can be seen from the overall low ToxPi values. However, a number of clusters of potential concern were observed both in terms of the samples with high bioactivity and cell type/phenotypes that were most frequently affected. For example, 6 of 46 tested samples exhibited high activity in iCell hepatocytes and about half of all samples were bioactive in iCell cardiomyocytes. Overall, there was a wide range of total bioactivity observed among 46 samples (Figure 2B) with sample HSC-12, collected at the mouth of Buffalo Bayou, exhibiting the highest ToxPi score. Even though samples from the Houston Ship Channel had an overall higher bioactivity as compared to other areas tested (Figure 2C), there was no significant difference in the means for each area because the ranges were largely overlapping.

### 3.3. Spatial Associations of Bioactivity and PAH Concentration Data in Sediment Samples

**Figure 3:**
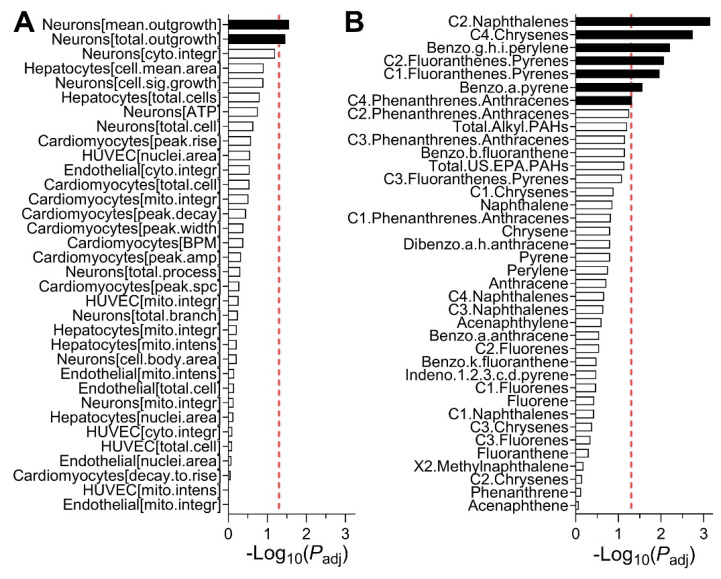
Spatial correlation among *in vitro* bioactivity (**A**) and PAH concentrations (**B**). Shown are adjusted *p* values (log_10_ scale) for spatial correlation of each parameter and a vertical dotted line represents *p_adj_* = 0.05 (false discovery rate) threshold. These *p* values were derived using a modified spatial correlation method as described in text.

Because we observed wide variability in bioactivity among samples in the entire study area, we tested if bioactivity and/or PAH concentrations were spatially associated. Specifically, we determined whether physical proximity among sampling sites was associated with the similarity of either bioactivity or PAH concentrations by using a statistical test of spatial association (a modified version of Mantel test [41]). We found that among the bioactivity phenotypes, only two endpoints from neuronal cell assays were significantly spatially correlated (Figure 3A). We note that stringent statistical correction was implemented in these analyses to guard against false positives. The lack of broad spatial association in bioactivity was in accord with finding in a previous study [11], indicating the sensitivity of the *in vitro* phenotypes to specific chemicals that may be present at each sampling location. However, a number of PAHs were significantly correlated in terms of their spatial distribution (Figure 3B), a finding that was also concordant with previous observations of the chemical contaminant “hot spots” of the chemicals in the same class during environmental sampling after Hurricane Harvey [11,44,45].

**Figure 4:**
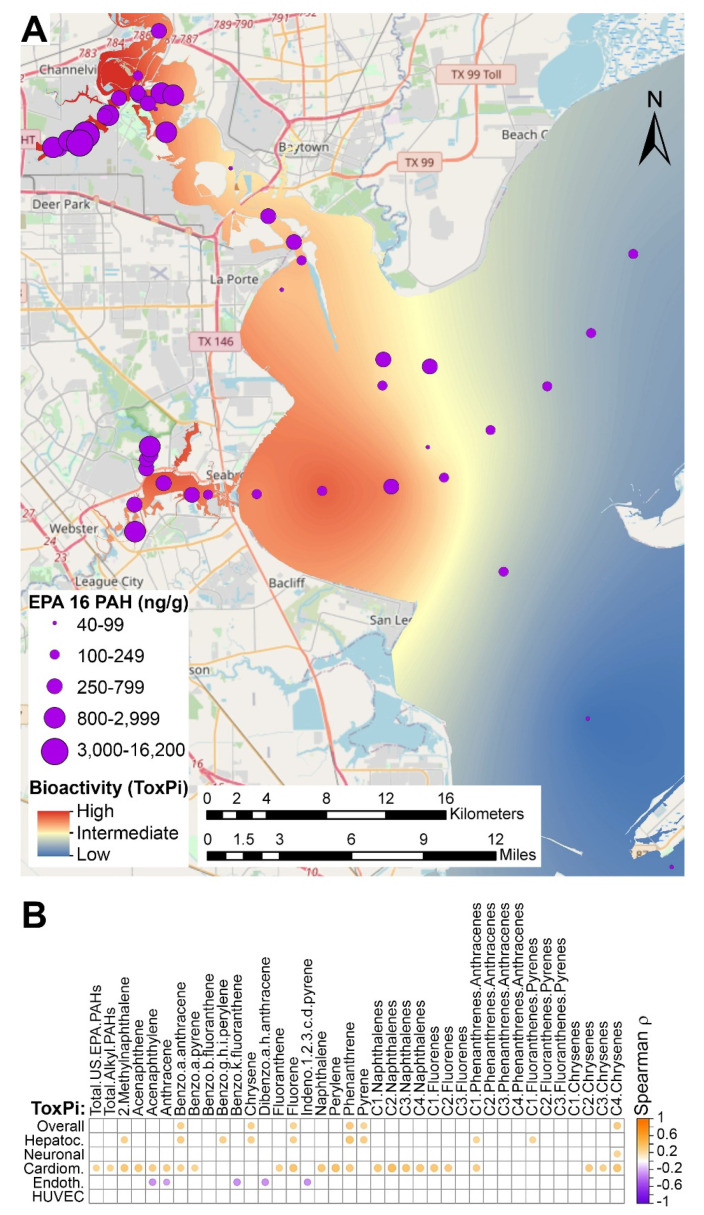
Correlation analysis between bioactivity and PAH content of the sediment samples. (**A**) Interpolation of the spatial patterns in bioactivity of samples based on all phenotypes from iCell cardiomyocytes. ToxPi values for this cell type were used to create the map that visualize bioactivity as a color gradient (red = highest, blue = lowest; see the legend inset for color gradient). Sampling locations are identified as purple dots and the cumulative concentration of 16 EPA priority PAH were used to scale each dot (see the legend inset for scaling). (**B**) Spearman correlation of all PAHs with total bioactivity in each cell type. Significant (*p*_adj_ < 0.05) correlations are shown as dots that are colored based on the *p* value as indicated in the color bar.

Next, we tested concordance between bioactivity and PAH concentrations. An example of spatial distribution of the bioactivity data (red-yellow-blue gradient) from iCell cardiomyocytes is shown in Figure 4A (see maps for other cell types as Supplementary Figure S1). Kriging analysis was used to interpolate to the entire sampling area based on the data from the individual testing locations. Sampling locations are shown as purple dots on the same map, with the size of each dot proportional to the total concentration of the 16 priority PAH in that sample. From this visualization, it is evident that the areas of greatest bioactivity in iCell cardiomyocytes are generally co-localizing with the highest PAH concentrations in the samples collected from Houston Ship Channel and Clear/Mud Lakes area. Thus, we examined whether *in vitro* bioactivity phenotypes and PAH concentrations correlated (Figure 4B). Most of the *in vitro* phenotypes (all of the phenotypes in HUVECs) did not correlate significantly with PAH values after adjustment for multiple comparisons; however, several of those, particularly from iCell cardiomyocyte and some from iCell hepatocytes, showed strong positive correlations. Positive correlation for the individual phenotypes is expected as it indicates that higher PAH concentration is associated with higher bioactivity (i.e., larger ToxPi value).

### 3.4. Predictions of Bioactivity and PAH Concentrations in Sediment Samples

It is traditionally expected that potential hazard (i.e., bioactivity) would be proportional to the exposure (i.e., chemical concentrations); however, while the bioactivity would be a product of cumulative effect of all chemicals in each sample, exposure assessment is typically conducted for each chemical class individually. In our study, detailed PAH data were available for these sediment samples. Therefore, by testing whether *in vitro* bioactivity data can be used collectively to infer PAH concentrations in these sediment samples, or vice versa, we could probe the strength of the associations between these two data types in addition to the spatial correlation analysis shown in Figure 4. This question is significant because both *in vitro* bioactivity and chemical analyses are time consuming and if these data streams are predictive of each other, more rapid assessment can be achieved by prioritizing sample analyses. Conversely, if there is little predictive power, it would imply that other chemicals could be present in samples and elicit bioactivity, suggesting that PAH analysis alone would not be sufficiently inclusive for risk management in this post-disaster scenario.

**Figure 5:**
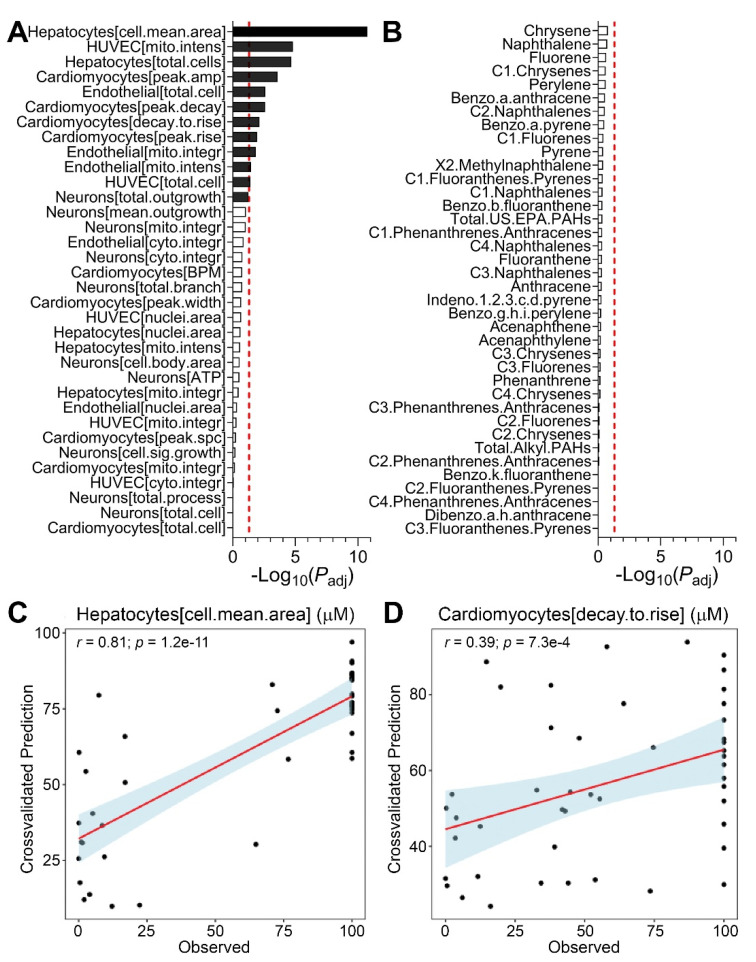
Cross-validated prediction of *in vitro* bioactivity and PAH concentrations. Significance of prediction for *in vitro* bioactivity data from PAH levels (**A**), or vice versa (**B**) is expressed as *p*_adj_ values (false discovery adjustment was performed using the Benjamini-Hochberg method). Significant results (*p*_adj_ < 0.05, vertical red dashed line) are shown as black bars. Also shown are illustrative cross-validated regression predicted values (y-axis) versus actual values (x-axis), for predicting (**C**) “cell.mean.area” phenotype in iCell hepatocytes, or (**D**) “decay.to.rise” phenotype in iCell cardiomyocytes. Dots are samples, red line is linear regression and blue shaded areas are 95% confidence intervals. Regression coefficients (*r*) and associated adjusted *p*-values are indicated.

To test this, we used a regression model with rigorous cross-validation to examine how well bioactivity data can be used to infer PAH concentrations (Figure 5A) or vice versa (Figure 5B). We found that about one third of the *in vitro* phenotypes can be inferred from PAH data (black bars in Figure 5A). For example, PAH concentrations were highly predictive of some of the bioactivity data with the strongest correlation (*r* = 0.81) for iCell hepatocytes “cell mean area” phenotype (Figure 5C). Even though the correlation coefficients for some of the bioactivity phenotypes were lower, they were still highly significant as in the example of iCell cardiomyocytes “decay.to.rise” phenotype (Figure 5D). Conversely, the bioactivity data were a poor predictor for PAH concentrations in the same samples, indicating that it is highly likely that there may be additional chemicals present in the sediments that may pose human health hazard. 

### 3.5. Risk Characterization and Site Remediation Scenarios from In Vitro Bioactivity or PAH data

**Figure 6:**
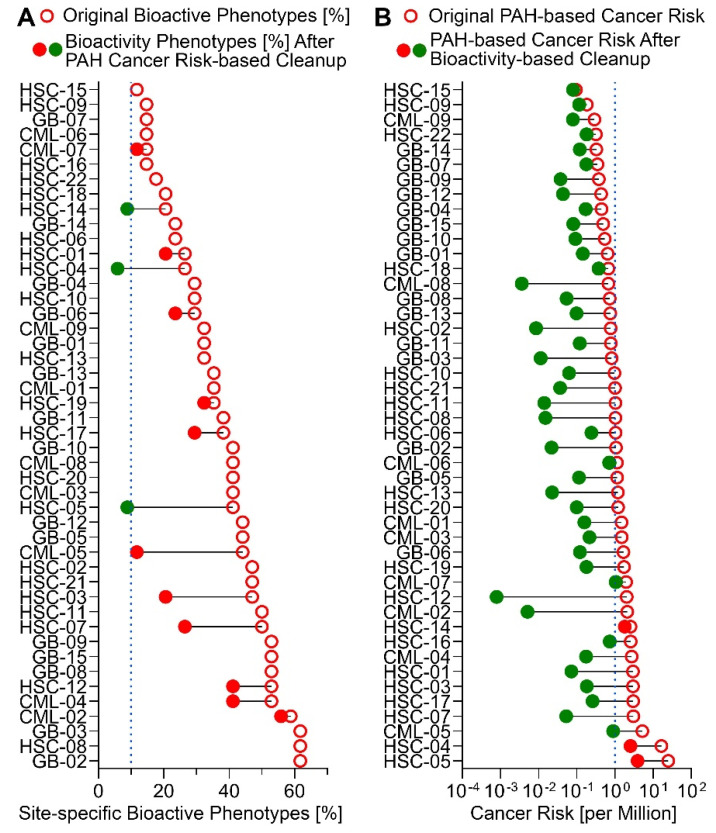
Risk Characterization at Baseline and Under Traditional and Bioactivity-based Remediation Goals. Shown are bioactivity (**A**) and cancer risk due to PAHs (**B**) at baseline (open red circles on the right of each dumbbell) and after traditional chemical-based (**A**) or bioactivity-based (**B**) remediation (closed circle on left of each dumbbell; green if below the remediation goal, red if still above the remediation target). The dotted lines represent the “target” remediation criteria (10% phenotypes active for bioactivity and “1 in a million” cancer risk for PAHs). Panel A shows that cleanup based on reducing traditional cancer risks is not protective with respect to remaining bioactivity, and results in substantial residual bioactivity across *in vitro* phenotypes. Panel B shows that cleanup based on reducing bioactivity is also protective of cancer risks for PAHs, reducing contamination in all but three sites to the residual risk values of < 1 in a million.

Finally, we evaluated the impact of using bioactivity data as an alternative basis for determining remediation goals, in comparison to tradition risk characterization using PAH concentrations. As shown in Figure 6, without remediation, all samples would exhibit bioactivity in at least 10% of phenotypes, but less than half of samples had PAH cancer risks greater than one in a million. Using PAH cancer risk as the basis for remediation, as would be traditionally the case, a substantial degree of bioactivity remains in the samples, with all but three samples having more than 10% of phenotypes bioactive, and more than 10 samples having at least 50% of phenotypes bioactive. By contrast, using bioactivity reduction to 10% of phenotypes as the basis for remediation, the resulting PAH cancer risks are <1 in a million for all except three samples, and < 1 in 100,000 for all samples (still acceptable risk, given the Superfund risk range of 10^−6^ to 10^−4^). This is because traditional risk assessment only “looks under the lamppost” and thus only results in risk mitigation for the “known” (measured) contaminants. Our analysis above showed that PAHs alone could not account for all observed bioactivity, and thus other bioactive chemicals must be present. Bioactivity-based remediation, on the other hand, in this case protects both against “known” risks such as PAHs, as well as “unknowns” that, while unidentified individually, collectively result in a bioactive signature indicative of their presence.

## 4. Conclusions

Hurricane Harvey made landfall on San Jose Island, Texas in August 2017 and resulted in extreme flooding in the coastal areas in Texas, including the city of Houston, a major population center in the United States, and the surrounding heavily industrialized areas. The event, from both rainfall and coastal flooding, resulted in broad redistribution of sediments in the Houston Ship Channel and Galveston Bay, including deposition of sediment known to be contaminated by a variety of chemicals [2,46,47] on shore in densely-populated residential areas. Previous studies documented redistribution of PAHs and other chemicals after Hurricane Harvey [4,5,6,48,49]. While the data on chemical contaminants in soil, water, or sediment samples would be typically used as a basis for risk management and remediation decisions [50], the distribution of the contaminants in affected areas is difficult to ascertain with precision over large areas of potential impact. In addition, there may be many chemicals present that are not evaluated [11,48]. Although *in vitro* test methods are routinely used to assess ecotoxicity of sediments [51], including in Galveston Bay [52], their utility for assessing human health risks from chemical exposures is a novel area of active study [7].

The broad disturbance of Galveston Bay sediment caused by Hurricane Harvey has established a new baseline of contamination, which have the potential to be redistributed again because of future storm events. Therefore, we posited that post-Harvey sediment samples can be used as a representation for contamination events after future disasters, and hypothesized that human *in vitro* cell-based assays may be highly informative, and complementary to traditional chemical analysis of PAHs, for risk management in situations that result in suspected redistribution of contaminants. Through hydrodynamic modeling we verified that a realistic major hurricane making landfall in the same area, a near certainty given the recent patterns of tropical storms in the Gulf of Mexico, will result in sediment redistribution from the Houston Ship Channel, Mud & Clear Lakes region, and Galveston Bay into densely-populated areas. Using sediment samples from these regions, we found a high range of bioactivity across samples, with the general trend of Houston Ship Channel > Mud & Clear Lakes > Galveston Bay. The spatial patterns of *in vitro* ecotoxicity in Galveston Bay [52] were qualitatively similar to those we found for human cell-based *in vitro* bioactivity, with greater potential hazards present in the Houston Ship Channel and Mud & Clear Lakes region, and lower farther out into the Galveston Bay. Interestingly, although most bioactivity phenotypes did not exhibit strong spatial correlations, some PAH concentrations were spatially associated. Moreover, while PAH concentrations were predictive of some bioactivity phenotypes, overall bioactivity data was a poor predictor of PAH concentrations, strongly suggesting that additional bioactive contaminants are present in addition to PAHs. This is further demonstrated in our risk characterization and evaluation of alternative remediation goals, where it is evident that setting cleanup levels based on PAH risks alone only may result in only modest reduction in overall hazard (i.e., bioactivity), whereas using bioactivity reduction as the criteria for cleanup will be protective against PAH cancer risks.

Overall, this study demonstrated the utility of *in vitro*, human cell-based assays to address the critical issue of “unknown” contaminants that may pose a human health hazard in a disaster scenario. Using post-Harvey sediment samples as a case study, our data shows that traditional chemical analysis of contaminants of concern (i.e., PAHs) may not account for the potential hazards (i.e., observed bioactivity). At the same time, *in vitro* bioactivity can be used as an alternative more relevant benchmark for rapid decision-making regarding cleanup levels that will be adequately protective with respect to “known” chemical exposures that may be present. We conclude that *in vitro* bioactivity is a comprehensive indicator of potential hazards and can be used as a new approach method (NAM) to inform risk management decisions on site cleanup during disaster events that may be associated with exposures to complex chemical mixtures.

## Data Availability

The data presented in this study are available in Supplemental Files.

## Supplementary Materials

The following are available online at https://www.mdpi.com/article/10.3390/ijerph182413378/s1, Supplementary Figure S1: Interpolation of the spatial patterns in bioactivity of samples based on all phenotypes from iCell hepatocytes, iCell neurons, iCell endothelial cells, and HUVECs. Supplementary Table S1: Sediment sample locations. Supplementary File S1: Regional Screening Levels (RSLs) calculation based on recreator exposure. Supplementary File S2: RSLs calculation based on resident exposure. Supplementary File S3: ToxPi scores for each sediment sample calculated from bioactivity POD values.

## Abbreviations

CR	cancer risk
DMSO	dimethyl sulfoxide
GB	Galveston Bay
HI	hazard index
HSC	Houston Ship Channel
HUVEC	human umbilical vein endothelial cell
iPSC	induced pluripotent stem cell
NAM	new approach method
PAH	Polycyclic Aromatic Hydrocarbon
PCB	polycyclic biphenyls
POD	point of departure
RSL	regional screening levels
ToxPi	toxicological priority index
