# Supplementary material for: Potential Human Health Hazard of Post-Hurricane Harvey Sediments in Galveston Bay and Houston Ship Channel: A Case Study of Using In Vitro Bioactivity Data to Inform Risk Management Decisions
Source: Int J Environ Res Public Health. 2021 Dec 19;18(24):13378. doi: 10.3390/ijerph182413378 (PMC8702027; doi:10.3390/ijerph182413378)
Supplement: Supplementary file 1 [file ijerph-18-13378-s001.zip › Supplementary Figure S1. Mapping based on bioactivities.pdf]

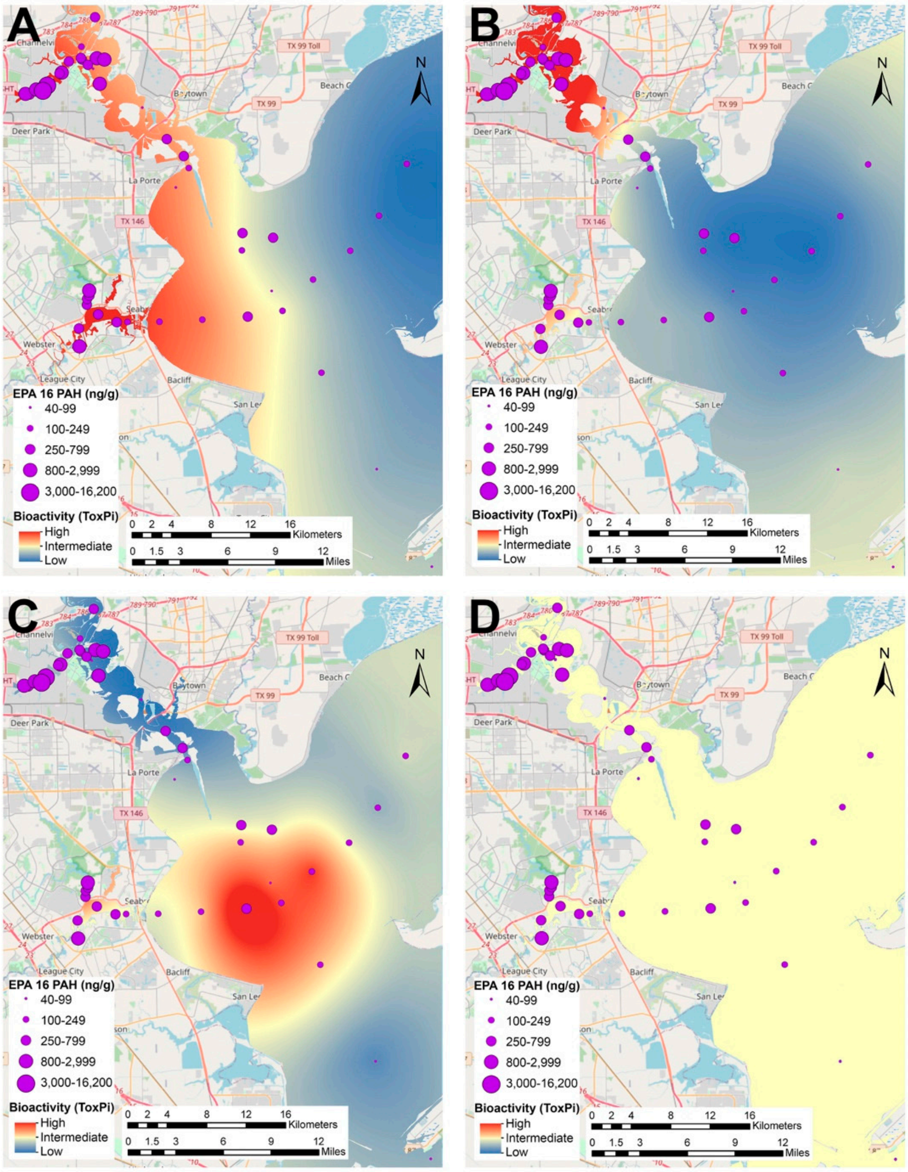

Supplementary Figure S1. Spatial patterns in bioactivity for samples based on the phenotypes from iCell Hepatocytes (A), iCell Neurons (B), iCell Endothelial cells (C), and HUVECs . ToxPi values for each cell type were used to create the map that visualizes (D)bioactivity as a color gradient (red=highest, blue=lowest; see the legend inset for color gradient). Sampling locations are identified as purple dots and the cumulative concentration of 16 EPA priority PAH were used to scale each dot.
